# Machine learning-based classification of histological subtypes of invasive breast cancer using MRI contralateral breast texture features

**DOI:** 10.1038/s41598-025-23498-7

**Published:** 2025-11-13

**Authors:** A. N. Nuzla, A. K. M. Nabeel, W. A. S. Nirmal, P. B. Hewavithana, M. L. Jayatilake

**Affiliations:** 1 Department of Radiography/Radiotherapy, Faculty of Allied Health Sciences, University of Peradeniya, Peradeniya, 20400 Sri Lanka; 2 Department of Radiology, Faculty of Medicine, University of Peradeniya, Peradeniya, 20400 Sri Lanka

**Keywords:** Invasive breast cancer, Invasive ductal carcinoma, Invasive lobular carcinoma, Magnetic resonance imaging, Machine learning, Cancer, Computational biology and bioinformatics, Medical research

## Abstract

Invasive Breast Cancer (IBC), encompassing Invasive Ductal Carcinoma (IDC) and Invasive Lobular Carcinoma (ILC), is the most prevalent cancer in women. This study aimed to develop a machine learning (ML) model for distinguishing between its histological subtypes (IDC and ILC) by analyzing glandular texture features from the contralateral breast. T1-weighted pre-contrast MRI images were sourced from the Cancer Imaging Archive, with image segmentation performed in 3D Slicer software, yielding a dataset of 2444 slices (1890 IDC, 554 ILC). First-order and GLCM texture features were extracted using MATLAB, and feature selection via ANOVA F-test revealed correlation (0.1233) and mean (0.5335) as the least significant features. Despite this, the initial model with all features achieved an accuracy of 0.9038, suggesting the importance of all extracted features. To address dataset imbalance, the SMOTE technique was applied, creating balanced training (80%) and testing (20%) subsets. Various ML algorithms were tested, and the Random Forest Classifier achieved the highest cross-validation scores for both SMOTE (0.8723 ± 0.0209) and original (0.8989 ± 0.0224) datasets. The final model achieved an accuracy of 91% on the original and 87% on the SMOTE dataset, revealing a comprehensive classification. The findings support early diagnosis and make an innovative contribution to the literature.

## Introduction

Breast cancer is one of the most prevalent malignancies affecting women globally, with a lifetime risk of 12.4%. In 2022, breast cancer was a leading cause of mortality worldwide, responsible for approximately 670,000 deaths and emerging as the most common cancer in 157 out of 185 countries^1^. Among its types, Invasive Breast Cancer (IBC) represents a significant clinical challenge. IBC includes localized breast cancer, which is confined to the breast, and metastatic breast cancer, characterized by the spread of cancer cells to distant parts of the body through the blood and lymphatic systems^2^. Within the staging system for breast cancer, in situ cancers are typically classified as Stage 0. Once invasive cancer is discovered, it is classified into stages 1 through 4 according to the Tumor, Nodes, and Metastasis (TNM) staging system. This staging relies on three clinical characteristics: the size of the primary tumor and whether it has invaded nearby tissue; the involvement of regional lymph nodes; and the presence or absence of metastasis to distant organs beyond the breast. Other elements that can influence staging include tumor grade, Human Epidermal Growth Factor Receptor 2 (HER2) status, estrogen receptor (ER) status, and progesterone receptor (PR) status^3^.

In breast cancer classification, the World Health Organization (WHO) and standard pathology guidelines categorize tumors based on histological type, which is determined through microscopic evaluation of tissue architecture. Invasive ductal carcinoma (IDC) is the most common form of invasive breast cancer and is characterized by duct-like morphological patterns, accounting for 80% of cases^4^. IDC originates in the milk ducts that channel milk from the glands to the nipple. A key clinical feature of IDC is the presence of a palpable “lump”. Genetic mutations, particularly in the BRCA1 and BRCA2 genes, are implicated in the development of IDC, leading to disrupted cell growth and division. Most IDCs exhibit ER and PR positivity^5^. In contrast, ILC represents a distinct histological subtype with lobular growth patterns, and it constitutes approximately 20% of invasive breast cancers. It typically arises from lobular carcinoma in situ (LCIS) in the milk-producing lobules, progressing into malignancy and infiltrating adjacent tissues. ILC may sometimes coexist with other types of breast cancer, such as ductal carcinoma in situ (DCIS) and IDC^6^. Unique to ILC is its diffuse growth pattern and linear infiltration of small, non-adherent cells into surrounding tissue^7^. This morphology stems from disrupted cell adhesion due to the absence of E-cadherin, a critical protein for cellular cohesion. Genetic alterations in the CDH1 gene underlie this deficiency, making the loss of E-cadherin a hallmark of ILC^8^. Both IDC and ILC are included under the histopathologic classification of breast cancer, alongside other variants such as tubular, mucinous, and medullary carcinomas.

### Risk factors of breast cancer

Breast cancer has several risk factors, including older age, personal and family history of breast or ovarian cancer, and genetic mutations such as those in BRCA1 and BRCA2. Additional contributors include hormonal influences (e.g., timing of pregnancy, early menarche, late menopause), lifestyle factors (e.g., weight, physical activity, alcohol consumption, smoking), and prior medical treatments like radiation therapy and hormone replacement therapy. Familial syndromes, such as Cowden, Li-Fraumeni, and Peutz-Jeghers also elevate risk^9^. Hereditary factors contribute to a small percentage (5–10%) of cases, whereas lifestyle and environmental factors account for a larger proportion (20–30%)^10^.

### Imaging techniques

Modern imaging modalities play a pivotal role in breast cancer detection and management. These include mammography, contrast-enhanced mammography, ultrasonography, magnetic resonance imaging (MRI), and others^11^. Among them, MRI demonstrates the highest sensitivity (94.6%) as a standalone modality, surpassing mammography and ultrasound^12,13^. The American College of Radiology (ACR) recommends annual MRI screening for women with a 20% or greater lifetime risk of developing breast cancer^14^. Advances in MRI technology, including improved 2D and 3D resolution, have expanded its utility in screening, diagnosis, and monitoring treatment response^15^.

### Texture analysis

Texture analysis is an innovative approach in medical imaging that characterizes spatial patterns of grey-level intensities within an image^16^. Originally developed for computer vision tasks, such as object classification and surface inspection, texture analysis has become indispensable in medical imaging^17^. It enables the quantitative assessment of tissue properties, aiding in the differentiation of normal and abnormal tissues. Texture features are categorized based on their statistical order^18^. First-order features are derived from grey-level histograms, including mean, median, and skewness. Second-order features are extracted from co-occurrence matrices, highlighting inter-pixel relationships^19^. In breast imaging, texture analysis holds promise for identifying parenchymal patterns and distinguishing between cancer subtypes^20^.

### Machine learning

Machine learning (ML) has revolutionized medical image analysis, enabling computers to “learn” from data and make predictions without explicit programming. ML approaches can be classified into three main categories: Supervised learning, Unsupervised learning, and Reinforcement learning^21^. Supervised learning constitutes a machine learning approach wherein the objective is to derive a function that accurately predicts outputs given corresponding inputs. This learning process relies on a dataset of labeled training examples, each comprising an input and its associated output^22^. On the other hand, unsupervised learning is trained on an unlabeled dataset^23^. In reinforcement learning, an autonomous agent learns to perform a task by trial and error, in the absence of any guidance from a user^24^. Supervised learning works best when the data is already highly predictive, such as when using “ground truth” labels. Commonly employed supervised learning classifiers include K-Nearest Neighbors (KNN), Logistic Regression (LR), Random Forest (RF), Naive Bayes (NB), Decision Trees (DT), and Support Vector Machines (SVM)^25^.

### Literature survey

In recent years, several significant studies have advanced this field by developing models to classify invasive breast cancer subtypes. Most of the research is based on analysing the cancer tumor using the contrast-enhanced data. We focus on a few of studies. In the year 2010, Holli et al., conducted a retrospective study to investigate the texture parameters of IDC and ILC in dynamic contrast enhanced magnetic resonance images in comparison to healthy tissue to characterize texture analysis findings of cancer. They selected the T1-weighted pre-contrast, two contrast enhanced series and their subtraction series. They achieved classification accuracy varying between 80% and 100% for all the employed classification methods, differentiating both cancerous and healthy breast tissue, as well as invasive ductal and lobular carcinoma^26^. In 2020, Yuan et al., carried out a study in China on differentiating tumor grades in breast invasive ductal carcinoma using texture analysis of MRI. They used Gobor wavelet analysis of the region of interest (ROI) for the tumor image to extract the texture properties. The feature subset was classified by using a SVM and its parameters were adjusted to achieve the best outcome. The highest accuracy was obtained in the range of 77.79%–81.94% for the prediction model after a five fold cross-validation. They concluded that there is a significant correlation between the grades of IDC and MRI^27^. In 2016, Sutton et al., conducted a retrospective study on breast cancer molecular subtype classifier that incorporates MRI features. Tumors were contoured on the fat-suppressed T1-weighted pre-contrast and three post-contrast images. They used a multiclass SVM for this study and a Leave-One-Out Cross Validation (LOOCV) approach. Each SVM fitted in the LOOCV process and generated a model with varying features. Eleven out of the top 20 ranked features were significantly different between IDC subtypes with *p* < 0.05. When the top nine pathologic and imaging features were incorporated, the predictive model distinguished IDC subtypes with an overall accuracy on LOOCV of 83.4%^28^.

The majority of studies reviewed in the literature rely on the analysis of tumors using contrast-enhanced data which often requires invasive procedures. Although the differences between IDC and ILC may be subtle, diagnosing ILC is notably more challenging due to its diffuse growth pattern. It resembles normal breast parenchyma in conventional imaging^29^.

This is where artificial intelligence (AI), especially advanced machine learning and deep learning techniques - can play a transformative role. Recent years have seen significant advances in AI-driven breast cancer detection and diagnosis across multiple imaging modalities. Notably, Fusion Siamese Networks have been developed to compare current and prior mammograms, emulating radiological comparison and reducing false positives^30^. Similarly, hybrid transformer-based models integrate prior and current images to improve representation learning for longitudinal mammogram analysis^31^. In addition, deep neural networks have been shown to enhance radiologists’ performance in breast cancer screening, achieving accuracy comparable to or superior to human experts^32^. Beyond mammography, AI techniques are increasingly applied to MRI, with emerging models delivering accurate breast cancer detection and explainable anomaly localization in both high- and low-prevalence settings^33^.

A comprehensive review by Chen et al. (2025) analyzed 181 AI-based studies published between 2020 and 2024, highlighting the strengths and applications of machine learning and deep learning in breast cancer imaging. Machine learning approaches using handcrafted features such as shape, texture, and intensity showed robustness and interpretability, particularly when paired with feature selection techniques. Deep learning methods automated feature extraction and demonstrated excellent performance on large datasets. The review also emphasized the utility of AI in dynamic contrast-enhanced MRI (DCE-MRI), enabling precise tumor volume measurement and fibroglandular tissue segmentation due to high spatial resolution^34^. Adam et al. conducted a review in 2023 that explored deep learning techniques for breast cancer detection with MRI, focusing on the capabilities of convolutional neural networks (CNNs) for classification, object detection, and segmentation tasks. While CNNs have shown promise in small-scale studies, there remains a need for large-scale, real-world evaluations to fully assess the performance of AI-driven breast MRI interpretation. Specifically, prospective and retrospective studies are necessary to validate the effectiveness of deep learning models in diverse clinical settings^35^. Recent retrospective studies further demonstrate AI’s potential in breast MRI. For example, Hirsch et al. (2025) presented a high-performance, open-source model trained on 6,615 breast MRI examinations, validated on both internal and external datasets, achieving state-of-the-art detection and localization performance. These studies collectively underscore the rapid progress in multimodal, longitudinal, and explainable AI approaches for breast imaging^36^. AI models excel at identifying non-obvious texture and tissue composition features across large image datasets, making them well-suited for analyzing the CLB. Recent advances in AI have shifted focus beyond just tumor detection to more nuanced applications such as risk stratification, prognostic modeling, and subtype classification. These developments directly support the motivation behind using the CLB for diagnostic purposes, as AI can harness faint patterns in normal-appearing tissue that may correlate with underlying malignancy or tumor subtype.

While most prior work focuses on tumor detection or classification in the affected breast, analyzing the contralateral (unaffected) breast provides a complementary perspective. Subtle glandular and texture patterns in the unaffected breast may reflect systemic or field effects associated with tumor biology, offering early indicators of molecular subtypes before overt lesions are detectable. This approach enables non-invasive prognostic assessment, enhances risk stratification for the contralateral breast, and captures broader tissue environment information, which can improve subtype prediction and contribute to personalized breast cancer management.

This study focused on developing a machine learning-based classification model to distinguish between different subtypes of invasive breast cancer using features extracted from breast MRI images. This was achieved by preprocessing and extracting relevant features from MRI images of patients diagnosed with invasive breast cancer, training machine learning algorithms on the extracted features, and evaluating the performance of the developed classification model using appropriate metrics such as accuracy, sensitivity, specificity, and Area Under the Curve-Receiver Operating Characteristic (AUC-ROC).

### Justification

Most studies rely on tumor data obtained through invasive methods, such as biopsy or contrast-enhanced imaging. Using contralateral breast data eliminates the need for invasive procedures, reducing patient discomfort, risks, and associated costs. The contralateral breast often serves as a reference for normal tissue. Leveraging its imaging data to identify subtle texture changes associated with invasive breast cancer introduces a novel diagnostic paradigm. This approach assumes that systemic changes in the breast due to malignancy may be reflected in the contralateral breast, even without visible tumor presence. ILC is particularly difficult to diagnose due to of its diffuse growth pattern, which often resembles normal tissue in conventional imaging. Texture analysis of the contralateral breast offers a new way to detect these subtle, often imperceptible changes. This method could identify imaging biomarkers that are not directly related to the tumor but instead reflect systemic or microenvironmental changes, potentially leading to earlier detection or more nuanced subtype classification. Many current studies depend on contrast-enhanced imaging, which can be contraindicated in some patients (e.g., those with kidney issues or allergic reactions). By using non contrast MRI data, this approach becomes more widely applicable. If successful, this approach could complement existing diagnostic workflows and provide additional insights into breast cancer biology, particularly in screening high-risk populations or evaluating contralateral breast health in patients with unilateral breast cancer. Existing literature largely focuses on the tumor and its surrounding tissues. Investigating contralateral breast data shifts the focus and contributes a fresh perspective to breast cancer imaging research, addressing a critical gap in current knowledge.

## Methods

This retrospective, quantitative study analyzed MRI data from female breast cancer patients to develop a machine learning-based model for classifying subtypes of IBC. This study employed a binary classification approach, with a supervised learning methodology used to develop the breast cancer subtype classification model.

### Data collection

The MRI images were obtained from the Cancer Imaging Archive website, which is managed by the Frederick National Laboratory for Cancer Research (FNLCR), in compliance with their data usage policies and restrictions. Breast MRI images of the patients from “Duke Breast-Cancer-MRI” dataset (https://www.cancerimagingarchive.net/collection/duke-breast-cancer-mri/) were collected through the archive in Digital Imaging and Communications in Medicine (DICOM) format, along with their age, type of breast cancer, tumor location, tumor grade (tubule), lymphadenopathy or suspicious nodes, and skin or nipple involvement. According to the selection criteria, only T1-weighted pre-contrast DICOM images were considered for this study.

### Image processing and region of interest selection

**Fig. 1:**
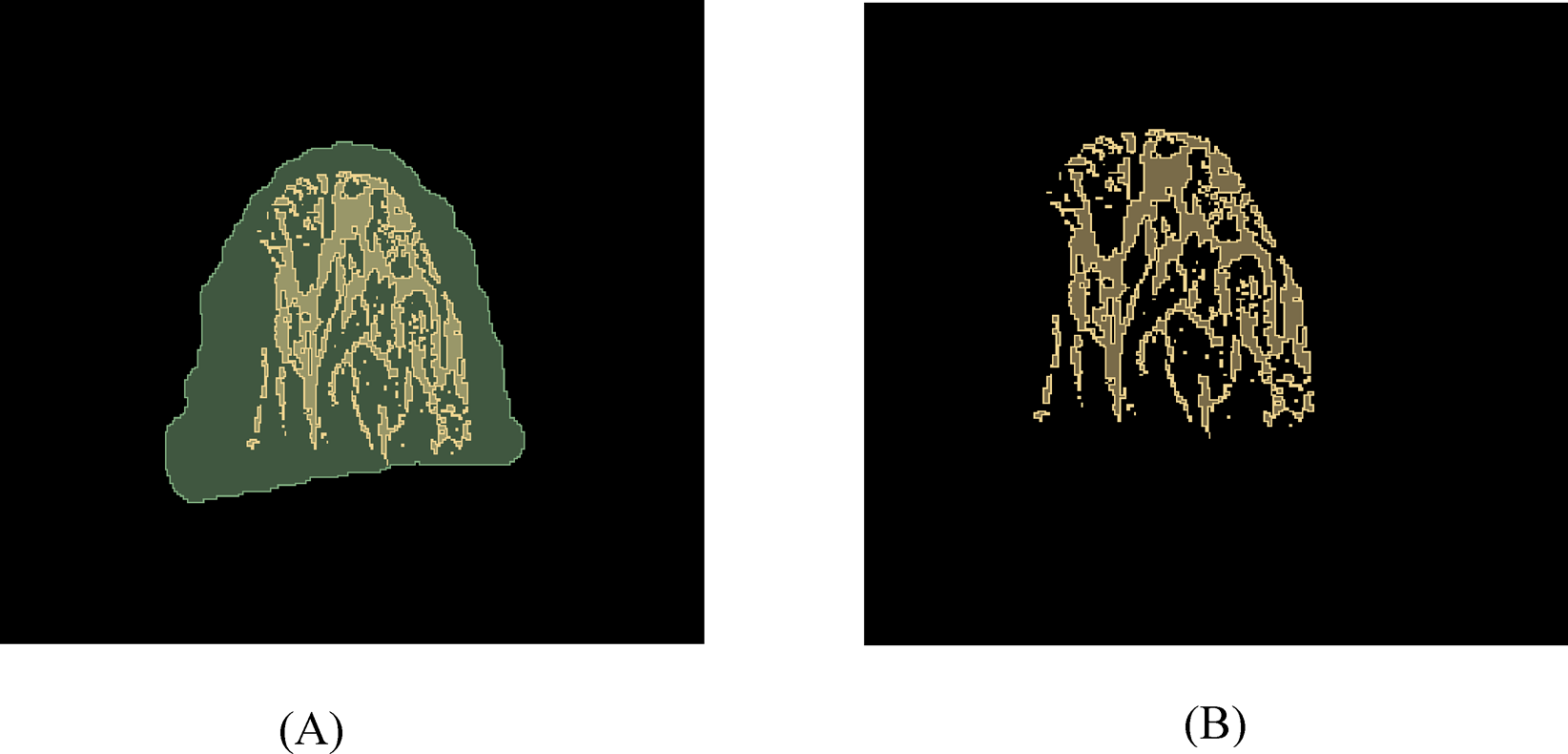
(**A**) shows the ROI for one slice of the glandular tissue of the CLB (axial plane). (**B**) shows the isolated glandular ROI for the same slice of the CLB (axial plane).

As the preprocessing step, DICOM viewer was used for initial viewing of the downloaded DICOM images. The ROI for the glandular area of the contralateral breast (CLB) was identified, with each slice marked accordingly. The glandular area of the CLB was segmented using the thresholding function of the 3D slicer application, generating the glandular segmentation mask. Quality control measures involved manual verification of segmentation results and ROI selection by the expert board-certified radiologist which ensured the accuracy and reliability. Glandular segmentation masks were applied to the original MRI slices of the breast images. Non-glandular tissue was masked out by multiplying the MRI slices with corresponding glandular masks, focusing the analysis on glandular regions (Fig. 1). In this manner, glandular ROI was isolated, and outlier slices containing ROI discontinuity were excluded. MATLAB (R2019b) was utilized to execute this process.

### Features extraction

The various texture descriptors capturing spatial patterns and heterogeneity within the glandular tissues were computed and tested for normality. First-order and GLCM texture properties were extracted from the glandular region of MRI breast images. First-order texture features are statistical descriptors of images based on the values of individual pixels. Extracted first-order statistical features included:

Mean: A measure of average intensity. This is the average grey-level value of the glandular tissue and can indicate the overall intensity.

1 $$\:Mean=\frac{1}{N}\sum\:_{i=1}^{N}{I}_{i}$$

Standard deviation: A measure of average contrast. This measures the variation of grey-level values within the glandular tissue and can provide information on the heterogeneity of the glandular tissue.

2 $$\:Standard\:deviation=\sqrt{\frac{1}{N}\sum\:_{i=1}^{N}{{(I}_{i}-\mu\:)}^{2}}$$

Skewness: This measure indicates the symmetry of the distribution.

3 $$\:Skewness=\frac{1}{N}\sum\:_{i-1}^{N}{\left(\frac{{I}_{i}-\mu\:}{\sigma\:}\right)}^{3}$$

Kurtosis: Measures the sharpness of the histogram of grey-level values.

4 $$\:Kurtosis=\frac{1}{N}\sum\:_{i=1}^{N}{\left(\frac{{I}_{i}-\mu\:}{\sigma\:}\right)}^{2}-3$$

Entropy: Measures the randomness or disorder of grey-level values considering all pixel values across the whole region.

5 $$\:Entropy=-\sum\:_{i-1}^{N}p\left({I}_{i}\right)log\left(p\right({I}_{i}\left)\right)$$

For the above Eqs. (1)-(5), N is the total number of pixels, $\:{I}_{i}$ is the intensity value of the i-th pixel, µ is the mean intensity, and σ is the standard deviation of the pixel intensities in the region.

Local entropy: A measure of the randomness or unpredictability of pixel values within a local neighborhood. It quantifies the complexity of the intensity distribution in a specific local area, rather than considering the entire region.

6 $$\:Local\:entropy=-\sum\:_{i-1}^{N}p\left({I}_{i}\right)log\left(p\right({I}_{i}\left)\right)$$

Local range: A measure of the difference between the maximum and minimum pixel intensities in the local region. The range is computed from the minimum and maximum intensity values, reflecting the spread of intensity values.

7 $$\:Range=max\left({I}_{i}\text{}\right)-min\left({I}_{i}\text{}\right)$$

Local standard deviation: A measure of the variation of pixel intensities within a local region or neighborhood of an image. It quantifies how much the pixel values deviate from the mean intensity of that specific local region.

8 $$\:Local\:standard\:deviation=\sqrt{\frac{1}{N}\sum\:_{i=1}^{N}{{(I}_{i}-{\mu\:}_{local})}^{2}}$$

For the above Eqs. (6)-(8), N is the number of pixels in the local neighborhood. $\:{I}_{i}$ is the intensity of the $\:\text{i}$ -th pixel in the local neighborhood, and $\:{\mu\:}_{local}\:$is the mean intensity of the pixels within the local neighborhood.

GLCM properties are a set of texture features that can be extracted using the GLCM, which captures the second-order statistical properties of the grey-level values in the glandular tissue. Extracted GLCM texture properties included:

GLCM contrast (CNT): GLCM contrast, also known as the sum of squares variance, measures the intensity difference between two neighboring pixels ($\:\text{i},\text{j}$) over the whole image. GLCM contrast becomes 0 for constant images ($\:\text{i}-\text{j}$), while the weights continue to increase exponentially as the difference of pixel intensities $\:(\text{i}-\text{j})$ increases. However, the edges, noise, or wrinkled textures within an image increase the contrast value.

9 $$\:CTN=\sum\:_{i,j=0}^{N-1}{P}_{i,j}\:{(i-j)}^{2}\:$$

GLCM correlation (COR): The linear dependency of grey levels on neighboring pixels of the image is measured by the GLCM correlation. When there is a linear and predictable relationship between the two pixels, the corresponding correlation increases. Therefore, the images with high correlation values express that there is high predictability of pixel relationship.

10 $$\:COR=\:\sum\:_{i,j=0}^{N-1}{P}_{i,j}\left[\frac{(i-{\mu\:}_{i})(j-{\mu\:}_{j})}{\sqrt{\left({{\sigma\:}_{i}}^{2}\right)({{\sigma\:}_{j}}^{2}})}\right]$$

GLCM Energy (ENR): The GLCM energy measures the uniformity of the grey level distribution of an image. An identically uniform distribution of grey levels in an image (window is very orderly) expresses 1 for GLCM energy and it becomes 0 for images that have an identically non-uniform distribution of grey levels. Here, GLCM energy uses each $\:{\text{P}}_{\text{i},\text{j}}$ value as a weight for itself in the calculation of GLCM energy.

11 $$\:ENR=\sum\:_{i,j=0}^{N-1}{{P}_{i,j}}^{2}$$

GLCM homogeneity (HOM): GLCM Homogeneity is the way of measuring the smoothness of distribution of grey levels within an image, which is inversely correlated with contrast.

12 $$\:HOM=\sum\:_{i,j=0}^{N-1}\frac{{P}_{i,j}}{1+{(i-j)}^{2}}$$

These 12 properties were extracted for all the samples and copied into an Excel file.

### Feature selection and model training

After feature extraction, a supervised learning method was applied to build a model for predicting types of IBC (IDC or ILC). A heat map was created to display the matrix’s values. The null values on the dataset were visualized in the heat map and then eliminated using a python method. The missing values in the data set were considered null, and were referred to as ‘not available data’ in the data set. The sample consisted of 2444 slices including 1890 IDC slices, and 554 ILC slices. The extracted feature values were normalized utilizing Python 3.7 to have zero mean and unit variance: where $\:\text{X}\text{n}$ is the feature normalized value, $\:\text{X}$ is the feature value and $\:\text{X}\text{m}\text{i}\text{n}$ and $\:\text{X}\text{m}\text{a}\text{x}$ are the minimum and the maximum values for the particular feature:

13 $$\:Xn=\:\frac{X\:-\:Xmin}{Xmax-Xmin}$$

This standardization process avoids the domination of features with high variance in the learning process. To select the most pertinent texture features, an analysis of variance (ANOVA) F-test was applied to assess their significance in distinguishing between ductal and lobular carcinomas. To address the class imbalance between IDC and ILC instances in the dataset, the SMOTE approach was employed. Subsequently, the original and SMOTE datasets were stratified and divided into training and testing subsets. The training subset comprised 80% of the data, while the remaining 20% was allocated to the testing subset. To determine the most promising algorithm for differentiating between ductal and lobular breast cancer, a tenfold cross-validation method was employed. Moreover, an under-sampling technique was also utilized in the same manner. The default parameters were examined in each of the algorithms including, K-Nearest Neighbor (KNN), Logistic Regression (LR), Random Forest (RF), Decision Tree Classifier (DTC), Gaussian Naive Bayes (GaussianNB), Support Vector Machines (SVM), and AdaBoost Classifier (ADA). Afterwards, a model for classifying breast cancer types was created by feeding the selected features into the Random Forest Classifier, as it was found to be the most pertinent algorithm to execute the study following the tenfold cross-validation test. The performance of the algorithm was evaluated using several parameters. The accuracy, precision, recall, F1 score, confusion matrix, and AUC-ROC measures were obtained with the set of parameters that produced the best model. Hyperparameter tuning for the Random Forest Classifier using a random grid search was performed to identify the ideal set of parameters in an effort to improve the accuracy of breast cancer type prediction. Grid search is a systematic method for hyperparameter tuning. It explores a predefined set of parameter values, making it suitable when the search space is relatively small and computational resources are available. This process involves systematically searching for the best combination of hyperparameters to optimize the model’s performance within the specified grid^37^.

The considered tunable hyperparameters of the algorithm were: n_estimators, max_depth, min_samples_split, min_samples_leaf, and bootstrap, and each hyper-parameter was tested within a predefined ranges of values (n_estimators: [50, 100, 200, 300], max_depth: [None, 10, 20, 30, 40], min_samples_split: [2, 5, 10], min_samples_leaf: [1, 2, 4], and bootstrap: [True, False]). The Receiver Operating Characteristic Curve (ROC) was used to estimate the performance of the developed classification model. In addition, the performance of the tuned classification model was assessed by observing accuracy, precision, recall, and F1 scores over the test set.

Accuracy expresses the proportion of all correct prediction from the total number of predictions made by the ML model:

14 $$\:Accuracy=\frac{TP+TN}{TP+TN+FP+FN}$$

Precision indicates the performance of a machine learning model by measuring the quality of positive predictions:

15 $$\:Precision=\frac{TP}{TP+FP}$$

Recall measures the correctly predicted positive cases out of all the positive individuals:

16 $$\:Recall=\frac{TP}{TP+FN}$$

The harmonic mean of precision and recall is represented by the F1 score:

17 $$\:F1=2.\frac{Precision.Recall}{Precision+Recall}$$

Where TP, TN, FP, and FN indicate True Positive, True Negative, False Positives, and False Negatives, respectively.

The performance of the ML model was evaluated again using the AUC-ROC, in addition to the previously mentioned performance evaluation metrics.

## Results

**Fig. 2:**
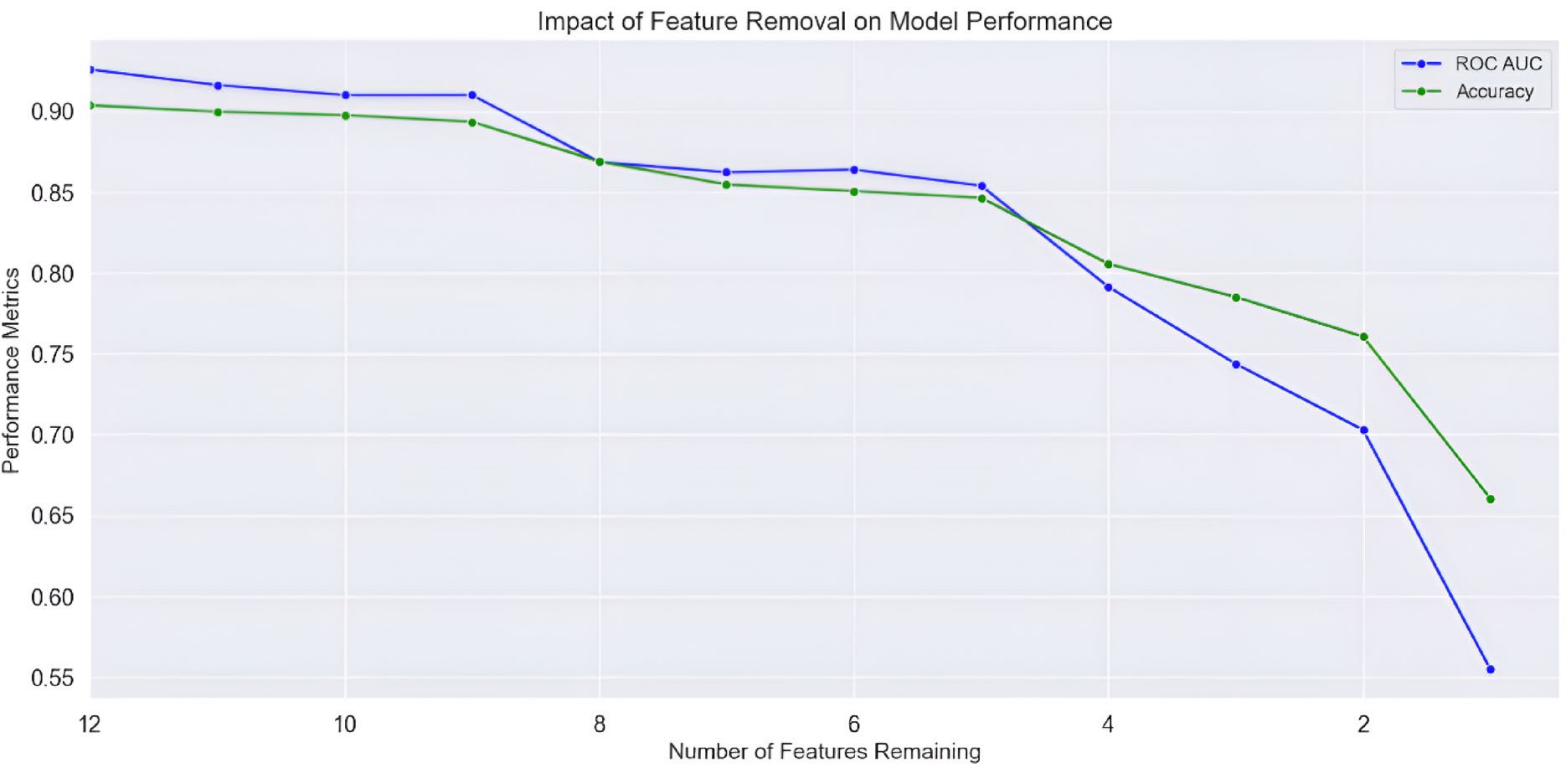
Corresponding model performance with ROC-AUC score (blue) and accuracy (green) as a function of feature removal (x-axis: number of features removed; y-axis: performance score).

The ANOVA F-test identified correlation (0.1233) and mean (0.5335) as the least significant features, while contrast achieved the highest score (87.4496). Sequential removal of features resulted in a steady decline in accuracy, with the initial model using all 12 features achieving the highest accuracy (90.38%). These results indicate that retaining all features is optimal (Table 1; Fig. 2).

**Table 1:** Results of the feature selection process, showing the sequential removal of features based on ANOVA F-test scores. The table includes the removed features, their corresponding F-test score, the number of remaining features, and the resulting model accuracy.

Removed feature	ANOVA F-test score	Number of features	Accuracy	*p*-value
Correlation	0.1233	12	0.9038	7.254574e-01
Mean	0.5335	11	0.8997	4.651769e-01
Entropy	4.2328	10	0.8977	3.975375e-02
Standard deviation	5.9237	9	0.8936	1.500902e-02
Energy	9.0595	8	0.8691	2.639950e-03
Local entropy	11.0821	7	0.8548	8.845887e-04
Kurtosis	32.9575	6	0.8507	1.058667e-08
Skewness	34.9334	5	0.8466	3.888354e-09
Homogeneity	53.9667	4	0.8057	2.765401e-13
Local standard deviation	69.9366	3	0.7852	1.015149e-16
Local range	74.2891	2	0.7607	1.191515e-17
Contrast	87.4496	1	0.6605	1.890132e-20

Among the algorithms tested, the Random Forest Classifier outperformed the others, achieving the highest accuracy and lowest variance for both the SMOTE (0.8723 ± 0.0209) and original (0.8989 ± 0.0224) datasets after ten-fold cross-validation. Based on these results, the Random Forest Classifier was selected to build the most accurate model for distinguishing between ductal and lobular breast cancer (Table 2).

**Table 2:** The mean cross-validation scores, standard deviation (SD), and the accuracy from different algorithms for the SMOTE and original datasets. The table illustrates the mean K-fold cross-validation scores the corresponding SDs acquired by each classification algorithm with and without the application of the SMOTE.

	Algorithm	Mean accuracy	Standard deviation (SD)	Accuracy as percentage
With SMOTE	KNN	0.6121	0.0340	61.21%
	LR	0.6440	0.0360	64.44%
	RF	0.8723	0.0209	87.23%
	DTC	0.8175	0.0197	81.75%
	GaussianNB	0.6914	0.0315	69.14%
	SVM	0.6833	0.0414	68.33%
	AdaBoost Classifier	0.7356	0.0225	73.56%
Without SMOTE	KNN	0.7418	0.0185	74.18%
	LR	0.7761	0.0034	77.61%
	RF	0.8989	0.0224	89.89%
	DTC	0.8653	0.0193	86.53%
	GaussianNB	0.7508	0.0193	75.08%
	SVM	0.7720	0.0029	77.20%
	AdaBoost Classifier	0.8183	0.0210	81.80%

**Fig. 3:**
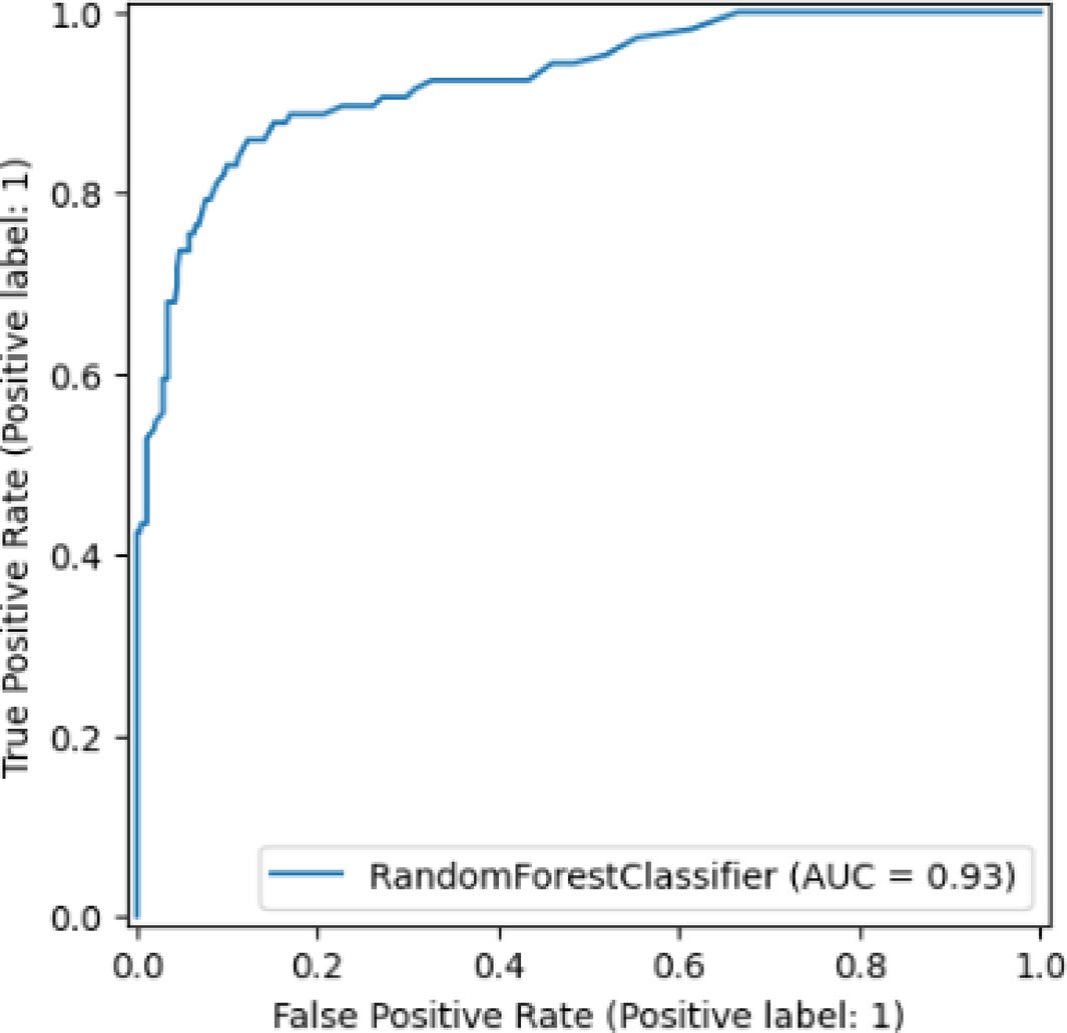
Binary class receiver operating characteristic (ROC) curve for the original dataset before hyperparameter tuning. The performance of tuned binary classification models is displayed in the ROC curve (x-axis: false positive rate; y-axis: true positive rate across a range of classification threshold) (range 1.00–0.00). The area under the receiver operating characteristic curve (AUC-ROC) score is 0.9259.

The generated model was able to predict the breast cancer type (ductal and lobular) with an overall accuracy of 90% after being trained using the Random Forest Classifier for the original dataset (Table 3). The area under the receiver operating characteristic curve (AUC-ROC) revealed a base model performance, with a score of 0.9259 (Fig. 3).

The considered tunable hyper-parameters of the algorithm were: n_estimators, max_depth, min_samples_split, min_samples_leaf, random_state, and bootstrap. Each hyperparameter was tested within predefined value ranges. After employing the grid search hyperparameter tuning, an increased overall accuracy of 91% was obtained for the original data set (Table 3). The best hyperparameters were n_estimators: 50, max_depth: 40, min_samples_split: 2, min_samples_leaf: 1, random_state: 42, and bootstrap: False. Furthermore, the developed tuned classification model correctly predicted 370 out of 383 invasive ductal carcinoma image slices, and 72 out of 106 invasive lobular arcinoma image slices (Fig. 5(A)).

**Table 3:** Performance of the developed machine learning model with and without hyperparameter tuning, before the SMOTE approach. The table illustrates the precision, recall, and F1-score for each invasive breast cancer category in both the base and tuned classification models using the original dataset. The invasive breast cancer categories 0 and 1 represent invasive ductal carcinoma and invasive lobular carcinoma, respectively.

	Category	Precision	Recall	F1 score	Support	Accuracy
Base model	0	0.92	0.97	0.94	383	90%
	1	0.85	0.68ara>	0.75	106	
Tuned model	0	0.92	0.97	0.94	383	91%
	1	0.85	0.69	0.76	106	

After applying the SMOTE approach, a significant change was demonstrated in the prediction of invasive breast cancer subtypes using the developed model with the Random Forest Classifier. The generated model was able to predict breast cancer types (ductal and lobular) with an overall accuracy of 86% without hyperparameter tuning, and 87% with tuning (Table 4). The best hyperparameters were n_estimators: 100, max_depth: none, min_samples_split: 5, min_samples_leaf: 1, and bootstrap: False.

**Table 4:** Performance of the developed machine learning model with and without hyperparameter tuning after applying the SMOTE approach. The table illustrates the precision, recall, and F1-score for each invasive breast cancer category in both the base and tuned classification models using the SMOTE dataset. The invasive breast cancer categories 0 and 1 represent invasive ductal carcinoma and invasive lobular carcinoma, respectively.

	Category	Precision	Recall	F1 score	Support	Accuracy
Base model	0	0.94	0.87	0.91	378	86%
	1	0.65	0.81	0.72	111	
Tuned model	0	0.93	0.89	0.91	378	87%
	1	0.69	0.78	0.73	111	

**Fig. 4:**
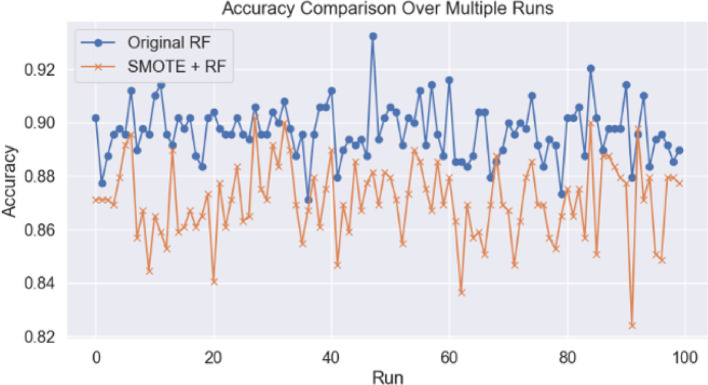
The figure presents a comparative analysis of bootstrap analysis, paired t-test, and Wilcoxon signed-rank test of classification accuracy between original and SMOTE datasets over 100 independent runs (Blue line: Original dataset; orange line: SMOTE dataset).

Due to the imbalance between IDC and ILC samples, an under-sampling technique was applied to improve predictions for ILC. The overall accuracy was measured at 85%, showing a slight deviation from previous findings. The area under the receiver operating characteristic curve (AUC-ROC) revealed the tuned model performance with a score of 0.9402. The developed tuned classification model correctly predicted 317 out of 378 invasive ductal carcinoma image slices, and 101 out of 111 invasive lobular carcinoma image slices (Figure 6).

**Fig. 5:**
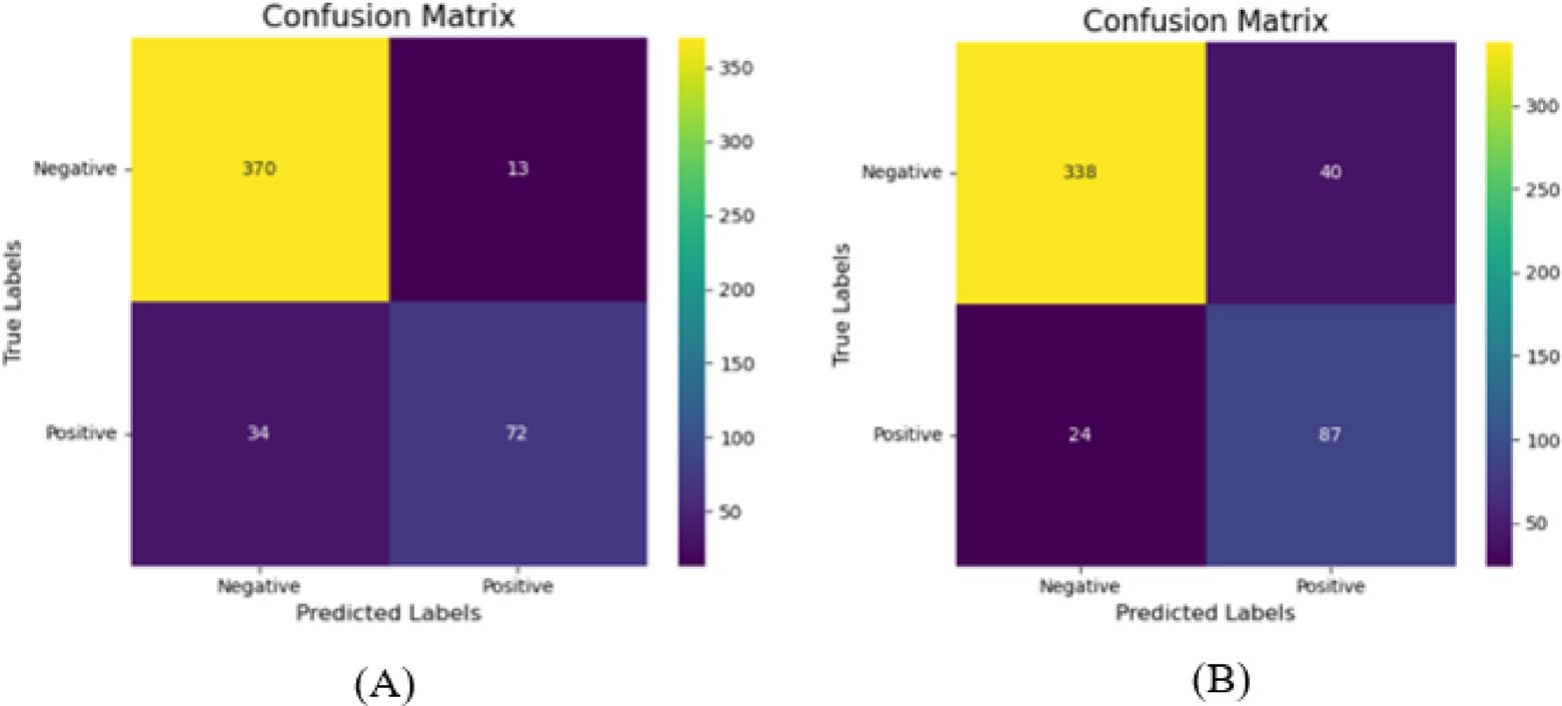
Confusion matrix illustrating the performance of the tuned classification model. The developed tuned classification model correctly predicted 370 out of 383 invasive ductal carcinoma image slices, and 72 out of 106 invasive lobular arcinoma image slices over the original dataset (A). It also correctly predicted 338 out of 378 invasive ductal carcinoma image slices, and 87 out of 111 invasive lobular arcinoma image slices over the SMOTE dataset (B).

**Fig. 6:**
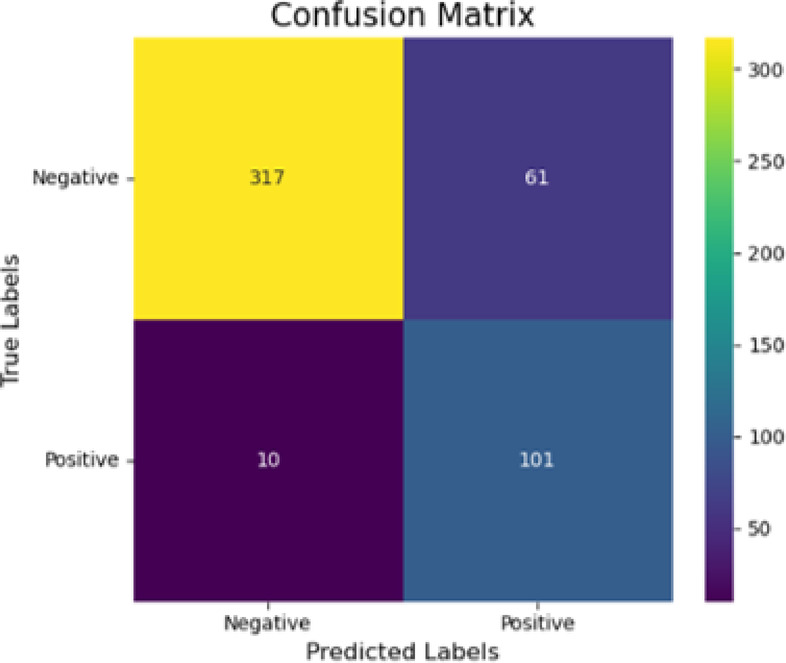
Confusion matrix illustrating the performance of the tuned classification model after applying the under-sampling technique. According to the confusion matrix, the developed tuned classification model correctly predicted 317 out of 378 invasive ductal carcinoma image slices, and 101 out of 111 invasive lobular carcinoma image slices.

## Discussion

There is a noticeable gap in the existing literature regarding robust methods for classifying IBC subtypes using contralateral breast MRI images. In this study, we proposed an automated, non-invasive technique to differentiate between IDC and ILC by analyzing statistical texture features, including first-order and GLCM (Grey-Level Co-occurrence Matrix) texture features. The texture features were extracted from each image slice using MATLAB software. The statistical analysis revealed that the categories contained unequal numbers of image slices, with 1890 slices for the IDC category and 554 for the ILC category. To mitigate the impact of this imbalance, the SMOTE technique was applied, resulting in the equalized sample sizes across categories, with both being adjusted to match the sample size of IDC, which had the largest representation in the dataset. A 90% of accuracy was obtained with the Random Forest Classifier which could classify the IDC and ILC over the original dataset after a ten-fold cross-validation experiment. However, after applying SMOTE, the accuracy scores of all algorithms degraded significantly. Despite this, the Random Forest Classifier secured the highest accuracy among all other employed classifiers (Table 2). The dataset with both non-equalized and equalized sample sizes for each invasive breast cancer subtype category was divided into train and test subsets (80% and 20% of the data, respectively). Random Forest Classifier was chosen as the most pertinent algorithm to execute the study, as it provided the highest accuracy score among all seven classifiers. Hyperparameter tuning for the Random Forest Classifier using random grid search was performed to identify the ideal set of parameters to improve the accuracy of invasive breast cancer type prediction. The accuracy, precision, recall, F1 score, confusion matrix, and AUC-ROC measures were obtained with the set of parameters that produced the best model. Comparing the precision, recall, and F1-score values of the base and the tuned model for each category, nearly the same values were obtained, with an improved overall accuracy (base model: 0.90 $\:<$ tuned model: 0.91) (Table 3), and AUC-ROC score (base model: 0.9259 $\:<\:$tuned model: 0.9274) demonstrated the high classification power of the developed model over the original dataset. Furthermore, there was an improved accuracy score observed for tuned model compared to the base model over the SMOTE dataset (base model: 0.86 $\:<$ tuned model: 0.87) (Table 4). A very low p-value (< 0.05) of paired t-test indicates that the difference in accuracy between the models is statistically significant. The Wilcoxon Signed-Rank test also supports this finding with a U-value of 5.0 and a p-value of 0.0000. The negative t-value (−17.151) shows that the original RF outperformed the SMOTE + RF in this comparison (Fig. 4). Our findings revealed a notable difference in predictive performance after hyperparameter tuning between original dataset and SMOTE dataset. The original dataset exhibited superior IDC prediction (original dataset: 370 out of 383 vs. SMOTE dataset: 338 out of 378) with 7.44% higher in prediction score, while the SMOTE dataset demonstrated a marked improvement in ILC prediction (original dataset: 72 out of 106 vs. SMOTE dataset: 87 out of 111) with 9.51% boosted prediction value (Fig. 5).

According to Alkhawaldeh IM, et al., the SMOTE technique generates synthetic samples for the minority class by interpolating between existing minority class samples. If these synthetic samples are too similar to the original minority class samples, the model may over-fit to these specific patterns, leading to a decrease performance on the majority class^38^. Improved ILC prediction over the SMOTE dataset indicates that balancing the dataset can enhance the model’s ability to detect ILC, which might be underrepresented in the original dataset. Mohammed et al., reported that the class imbalance is a prevalent challenge in binary classification problems, where the distribution of instances between the two classes is significantly skewed. This can lead to inaccurate classification models, particularly for the minority class, as it is often misclassified. Moreover, the limited data available for the minority class can restrict model training, resulting in under-fitting or over-fitting^39^. By increasing the representation of minority classes, SMOTE helps machine learning models learn the patterns and characteristics of the minority class more effectively. This often leads to improved classification accuracy, especially for the minority class^40^.

Following model tuning, the under-sampling technique was found to be the most effective in predicting ILC, correctly identifying 101 out of 111 slices and surpassing the performance of the other two methods with the ILC prediction accuracy of 90.99% (Fig. 6). According to Yen et al., the SMOTE technique generates synthetic minority class samples independently of the majority class, which can potentially lead to overgeneralization. Due to the significant discrepancy in the number of samples between the majority and minority classes in imbalanced datasets, under-sampling techniques aim to mitigate this imbalance by reducing the number of instances belonging to the majority class^41^.

This investigation, specifically examining the contralateral breast yielded highly accurate results in differentiating IDC and ILC across the three investigated approaches. When comparing the results of this study to those of Holli et al. (2010), their classification accuracy ranged from 80% to 100% across all employed methods for distinguishing between IDC, ILC, and healthy breast tissue. However, it is important to note that their approach differs from ours. In their methodology, they utilized T1-weighted pre-contrast images, two contrast-enhanced series, and their corresponding subtraction series.

While this research represents a promising step forward, several limitations and directions remain. A significant concern is that this study assumed the contralateral breast to be entirely healthy. While the primary breast tumor often commands attention, the contralateral breast is frequently neglected. Conversely, it is well-established that women with a history of invasive breast cancer have an increased risk of developing a secondary tumor in the healthy breast^42^. Initial research findings indicate that MRI may identify occult contralateral breast cancers in approximately 5% of women who have recently been diagnosed with breast cancer^43^. Thus, it is important to acknowledge the increased risk of developing breast cancer in the contralateral breast, which could introduce potential biases in the analysis. This challenge can be addressed by conducting a comparative analysis of breast MRI images obtained from a healthy population. A comparison of contralateral-only, ipsilateral-only, and bilateral breast inputs would be a valuable direction to assess the added benefit of incorporating contralateral breast texture features into the classification pipeline.

Despite the fact that this study highlights the potential of conventional machine learning for classifying breast cancer subtypes using contralateral breast texture features, future work could explore advanced deep learning models, such as Convolutional Neural Networks (CNNs) and Vision Transformers (ViTs), which excel at learning complex image features, but require large-scale, well-annotated datasets, to achieve generalizability and clinical robustness - resources that are currently limited in public breast MRI repositories, particularly for ILC cases. To bridge this gap, future studies could investigate transfer learning from large general-domain datasets, semi-supervised learning to leverage unlabeled data, or model distillation approaches that retain performance while improving explainability. Additionally, combining radiomics with deep learning features could offer a balanced, robust approach. As imaging datasets expand, these strategies may enhance clinical applicability and diagnostic accuracy.

## Conclusion

The findings suggest that the extracted texture features from contralateral breast including first-order statistical texture features—such as mean, standard deviation, skewness, kurtosis, entropy, local entropy, local range, and local standard deviation, and GLCM texture features—such as contrast, correlation, energy, and homogeneity may serve as valuable biomarkers for differentiating histological breast cancer subtypes, specifically IDC and ILC. Considering the scope of machine learning applications and the specific focus on the contralateral breast, the obtained accuracy levels are deemed acceptable. The findings are expected to contribute significantly to improving breast cancer screening in such settings. Future research should focus on integrating the obtained results into clinical workflows with validation required across diverse populations. In conclusion, this study contributes to the growing field of ML in medicine by showcasing the potential of ML techniques to achieve the classification of IDC and ILC. By addressing the identified limitations and pursuing future research directions, the full potential of ML can be unlocked to revolutionize healthcare and improve patient outcomes.

## Data Availability

Freely available online dataset from the cancer imaging archive. (https://www.cancerimagingarchive.net/collection/duke-breast-cancer-mri/).

## Abbreviations

GLCM: Grey level co-occurrence matrix
IBC: Invasive breast cancer
IDC: Invasive ductal carcinoma
ILC: Invasive lobular carcinoma
MATLAB: Matrix laboratory
ML: Machine learning
MRI: Magnetic resonance imaging
ROC: Receiver operating characteristic
SMOTE: Synthetic minority oversampling technique
